# Qualities Important in the Selection of Chief Residents

**DOI:** 10.7759/cureus.7580

**Published:** 2020-04-07

**Authors:** Joseph Turner, Megan Litzau, Josh Mugele, Katie Pettit, Elisa J Sarmiento, Aloysius Humbert

**Affiliations:** 1 Emergency Medicine, Indiana University School of Medicine, Indianapolis, USA; 2 Emergency Medicine, Mercy Hospital, St. Louis, USA; 3 Emergency Medicine, Northeast Georgia Medical Center, Gainesville, USA

**Keywords:** chief resident

## Abstract

Background

Chief resident selection occurs by numerous methods. Chief residents also fulfill multiple roles, requiring a broad skill set. However, there is little literature on which qualities various stakeholders value in chief resident selection. The objective of this study was to identify the qualities that residents and faculty believe are important for chief residents.

Methods

Following a literature review, educational experts conducted a multi-institutional survey that asked participants to name the qualities they felt were most important in chief residents and to rank-order a predefined list of 10 qualities. Associations were calculated between rank-order and participant age, gender, institutional position, and history of serving as a chief resident.

Results

The response rate for the survey was 43.9% (385/877). Leadership, organization, and communication skills were named by all participants among the most common responses. Residents additionally named approachability, advocacy, and listening skills among their most valued qualities, whereas faculty named strong clinical skills and integrity. Dependability and trustworthiness were the most valued qualities in the rank-order list, whereas strong clinical skills and self-reflection were the least valued. Females valued the ability to manage multiple demands more whereas males valued dependability more. The faculty valued strong clinical skills more than residents.

Conclusion

A variety of qualities are seen as being valuable in chief residents. Additional research is needed to understand what qualities are associated with effective chief resident performance.

## Introduction

The chief resident (CR) role in graduate medical training serves as a leadership role for senior residents and is traditionally seen as a stepping-stone to leadership roles in their future career whether in academic or community medicine. The role and responsibilities of CR vary depending on medical specialty and institution. Additionally, in some specialties, such as surgery, all residents in their last year of training are designated CRs, whereas, in others, the chief year is an extra postgraduate year [1]. In many cases, a percentage of senior residents are selected to serve as CR. Depending on the nature of the role and responsibilities (i.e., the job description), a variety of personal characteristics or qualities may be desirable for individuals in that role.

In emergency medicine (EM), the CR is typically chosen in an election-style process, commonly by some combination of program directors (PDs), faculty, and residents [1]. CRs have a wide variety of responsibilities and duties, including administrative duties (such as creating schedules), acting as the liaison between residents and PDs, role-modeling clinical behavior, informal mentoring of fellow residents, and educating junior residents and medical students (Abstract: Playe, Squillante, Durkin, and Brennan. Chief Residency in Emergency Medicine. SAEM Annual Meeting. 1998) [1-5]. It is intuitive that different qualities will be valued differently by various stakeholders selecting CRs and different qualities will vary in importance based on specific responsibilities. For example, a PD may value honesty and integrity in a potential CR, whereas residents might value fairness and attention to detail in a CR who will be creating a schedule.

To our knowledge, there is no literature that examines the perceived importance of various qualities when selecting CRs in EM residency programs. With this in mind, we surveyed residents and faculty at a number of EM institutions in order to assess the relative importance of various qualities when selecting CRs.

## Materials and methods

Participants and setting

In addition to the investigators’ home institution, we chose 10 institutions to participate in the study. Institutions were selected to represent a variety of EM residencies reflecting a diversity of program geography, size, and duration of training. Two programs declined to participate, leaving a total of nine programs in the study. We distributed the survey to all EM residents and faculty at the participating programs.

Survey development and distribution

The electronic survey (Appendix A) comprised three sections displayed on three separate pages. Section one surveyed participant demographics. Section two asked participants to identify the first, second, and third most important qualities that a CR should possess.

Section three asked the participant to rank 10 qualities from most important to least important from a provided list. We created this 10-item list in conjunction with a medical librarian and an expert in medical education by reviewing literature pertaining to CRs, academic medicine, and general leadership, generating an initial list of 71 qualities we thought might be important in a CR [2-13]. Then, we iteratively categorized, batched, and narrowed the terms to a list of 10 items that were widely represented in the literature, represented a broad range of quality types, and were felt to be important by all team members.

The survey was conducted using Qualtrics (Provo, UT) software. It was initially sent in February 2018 to a contact person at each institution, who then distributed it to faculty and residents at that institution, with a single reminder sent one month later.

Analysis

Free-text responses from section two of the survey were reviewed by two study investigators. Terms that were similar, particularly when they contained the same root (eg “communication skills” and “good communicator”) were combined when agreed upon by both investigators. We then sorted by frequency of occurrence for: 1) all participants, 2) resident participants, and 3) faculty participants. We calculated the difference in frequency between resident responses and faculty responses. We also looked at the frequency of responses among PDs/assistant PDs (APDs).

Variable definitions

For analysis, age was categorized as < 35 years old and ≥ 35 years old. The institutional position was categorized as faculty or resident. Other variables included gender (male/female) and whether or not the respondent had served as a CR (yes/no).

Statistical analysis

For rank-order analysis, we compared demographic characteristics with CR qualities using the Wilcoxon Signed-Rank test. We chose this test due to the non-normal distribution and rank-ordered attributes of the data. Multivariable linear regressions with the generalized linear model (GLM) procedure in SAS (SAS Institute, Cary, NC) was created using the variables observed to be statistically different (p = < 0.05) on the bivariate analysis, including open to multiple viewpoints, strong clinical skills, effectively manage demands, dependable, good listener, and trustworthy adjusted for age, gender, race, institutional position, and whether or not the respondent had served as CR. We used PROC GLM to report the estimates for the categorical independent variables in the models. We performed all statistical analyses using SAS Version 9.4.

## Results

The overall response rate for the survey was 43.9% (385/877), with a similar response rate among faculty (184/419 = 43.9%) and residents (201/458 = 43.9%). Of the 877 responses, 816 included all response elements, whereas 61 included only demographic data and the rank-order without free-text responses. The demographic data for the respondents are in Table 1.

**Table 1:** Demographics PD: program director; APD: assistant program director

Characteristic	All, No. (%) (N=385)
Position	
R1 resident	59 (15.3)
R2 resident	64 (16.6)
R3 resident	78 (20.3)
PD/APD/Former PD	18 (4.7)
Other faculty/fellow, < 5 years out	50 (13.0)
Other faculty/fellow, >5 years out	116 (30.1)
Gender	
Female	165 (42.9)
Male	213 (55.3)
Other/declined comment	7 (1.8)
Age	
<26	1 (0.3)
26-30	140 (36.4)
31-35	103 (26.8)
36-40	51 (13.2)
41-45	30 (7.8)
46-50	28 (7.3)
51-55	14 (3.6)
>55	16 (4.2)
Other/declined comment	2 (0.6)
Current/Former chief resident?	
Yes	128 (33.2)
No	257 (66.8)

Free-text responses

The most frequently occurring free-text responses are listed in Table 2. Organization was the most commonly named quality by all respondents, as well as from residents. Leadership was the most commonly named quality by faculty. Qualities with the largest frequency difference between the resident and faculty are listed in Table 3.

**Table 2:** Most common free-text responses (N = number of responses) Multiple qualities with similar frequency were clustered following the displayed qualities.

Residents	Faculty	All Respondents
Organized (51)	Leader/Leadership/Leads by Example (52)	Organized (91)
Approachable (39)	Organized (40)	Leader/Leadership/Leads by Example (80)
Leader/Leadership/Leads by Example (28)	Strong clinical skills (37)	Strong clinical skills (55)
Communication skills/Good communicator (24)	Communication skills/Good communicator (27)	Communication skills/Good communicator (51)
Hard working/Good work ethic (18)	Integrity (21)	Approachable (44)
Strong clinical skills (18)	Hard working/Good work ethic (13)	Hard working/Good work ethic (31)
Fair(13)		Integrity (30)
		Fair (24)

**Table 3:** Free-text responses with >5 response difference between groups

Resident Frequency > Faculty		Faculty Frequency > Resident	
Quality	Difference	Quality	Difference
Approachable	34	Leader/Leadership/Leads by Example	24
Organized	11	Strong clinical skills	19
Advocate	8	Integrity	12
Listening ability/Good listener	8	Resilient	6

Table 4 displays the qualities that respondents most frequently identify as most important in the free-text response section. Residents and faculty both cited leadership most frequently as the top quality.

**Table 4:** Most Important Quality, Free-text Responses (N = number of responses) Multiple qualities with similar frequency were clustered following the displayed qualities

Residents	Faculty	All Respondents
Leader/Leadership/Leads by Example (15)	Leader/Leadership/Leads by Example (38)	Leader/Leadership/Leads by Example (53)
Approachable (14)	Strong clinical skills (12)	Communication skills/Good communicator (19)
Communication skills/Good communicator (11)	Organized (10)	Organized (19)
Organized (9)	Integrity (9)	Approachable (15)
Reliable (7)	Communication skills/Good communicator (8)	Integrity (14)
		Strong clinical skills (14)

When comparing responses from participants who had served as CR versus those who had not been CR, both groups named leadership most frequently as the highest quality, with a response frequency of 17.98% among chiefs and 10.11% among non-chiefs. One other quality was identified as most important at a frequency greater than 5% among the chief cohort: strong clinical skills was cited by 5.47% of chiefs vs. 0.39% of non-chiefs. Organization was the only additional quality identified as most important by more than 5% of the non-chiefs (5.84% among non-chiefs vs. 3.91% among chiefs).

The PD/APD respondents named a total of 36 different qualities, with seven qualities (organization, communication, fairness, honesty, integrity, leadership, and teaching/education interest) receiving more than one response [2-4]. There were 13 different qualities identified as most important by this group, with only two qualities (organization and integrity) receiving more than one response in the most important slot [2-3].

Quality rank ordering

The rank-order of the 10 pre-chosen qualities, along with the median rank position, is in Table 5. Dependable and trustworthy were tied for the most important quality, while self-reflective and strong clinical skills were tied for the least important.

**Table 5:** Order of ranked qualities (median rank position)

T-1. Dependable (3.0)
T-1. Trustworthy (3.0)
3. Effectively manages multiple demands (4.0)
T-4. Equitable/Fair (5.0)
T-4. Positive attitude (5.0)
T-6. Effectively conveys ideas (6.0)
T-6. Good listener (6.0)
T-6. Open to multiple viewpoints (6.0)
T-9. Self-reflective (9.0)
T-9. Strong clinical skills (9.0)

Tables 6-9 display a comparison of the rank-order of the qualities with age, gender, position (resident vs. faculty), and history of serving as CR. After multivariable adjustment, there was a statistically significant association between female gender and ranking the quality, managing multiple demands highly, and between male gender and ranking dependability highly. There was also a significant association between being faculty and ranking strong clinical skills highly. There were no statistically significant associations between age or history of serving as CR and rank-order on multivariate analysis, though there was a strong trend of respondents who had served as CR ranking trustworthy and strong clinical skills more highly.

**Table 6:** Analysis of age with order of ranked qualities † Median (IQR); *Estimated using Wilcoxon Signed Rank Test. ** Adjusted for age, gender, and institutional position, and estimated using GLM GLM: generalized linear model

Age	<35 years	≥ 35 years	P-Value, Bivariate*	P-Value, Multivariable Adjustment**
Chief resident qualities^†^				
Effectively manage multiple demands	4.0 (2.0-7.0)	4.0 (3.0-7.0)	0.3571	
Dependable	2.0 (2.0-4.0)	3.0(2.0-5.0)	0.1552	
Effectively convey ideas	6.0 (5.0-8.0)	6.0 (5.0-7.0)	0.2410	
Equitable/ Fair	4.0 (3.0-6.0)	5.0 (3.0-7.0)	0.1383	
Good listener	6.0 (4.0-8.0)	6.0 (4.0-8.0)	0.4443	
Open to multiple viewpoints	5.0 (4.0-7.0)	6.0 (4.0-8.0)	0.0045	0.4204
Positive attitude	5.0 (3.0-8.0)	5.0 (2.0-8.0)	0.4985	
Self-reflective	9.0 (8.0-10.0)	9.0 (8.0-10.0)	0.5787	
Strong clinical skills	9.0 (7.0-10.0)	7.5 (3.0-10.0)		0.6716
Trustworthy	3.0 (7.0-10.0)	3.0 (2.0-6.0)	0.8987	

**Table 7:** Analysis of gender with order of ranked qualities † Median (IQR); *Estimated using Wilcoxon Signed Rank Test. ** Adjusted for age, gender, and institutional position, and estimated using GLM GLM: generalized linear model

Gender	Female		Male	P-Value, Bivariate*	P-Value, Multivariable Adjustment**
Chief resident qualities^†^					
Effectively manage multiple demands	4.0 (1.0-5.0)	5.0 (3.0-7.0)			0.0003
Dependable	3.0 (2.0-5.0)	2.0(1.0-4.0)		0.0010	0.0028
Effectively convey ideas	6.0 (5.0-8.0)	6.0 (4.0-8.0)		0.8334	
Equitable/ Fair	4.0 (3.0-7.0)	5.0 (3.0-6.0)		0.5984	
Good listener	6.0 (4.0-8.0)	6.0 (4.0-7.0)		0.1649	
Open to multiple viewpoints	5.0 (4.0-7.0)	6.0 (4.0-8.0)		0.4858	
Positive attitude	5.0 (3.0-8.0)	5.0 (3.0-8.0)		0.2554	
Self-reflective	9.0 (8.0-10.0)	9.0 (8.0-10.0)		0.9230	
Strong clinical skills	9.0 (4.0-10.0)	9.0 (5.0-10.0)		0.6463	
Trustworthy	3.0 (2.0-6.0)	3.0 (2.0-5.0)		0.7979	

**Table 8:** Analysis of grouped institutional position with order of ranked qualities † Median (IQR); *Estimated using Wilcoxon Signed Rank Test. ** Adjusted for age, gender, and institutional position, and estimated using GLM GLM: generalized linear model

Institutional Position	Faculty	Resident	P-Value, Bivariate*	P-Value, Multivariable Adjustment**
Chief resident qualities^†^				
Effectively manage multiple demands	4.0 (2.0-6.0)	5.0 (2.0-7.0)	0.2825	
Dependable	3.0 (2.0-5.0)	2.0 (2.0-4.0)	0.0547	
Effectively convey ideas	6.0 (5.0-8.0)	6.0 (4.0-8.0)	0.9735	
Equitable/ Fair	5.0 (3.0-7.0)	4.0 (3.0-6.0)	0.0534	
Good listener	6.0 (5.0-8.0)	6.0 (3.0-7.0)	0.0016	0.0056
Open to multiple viewpoints	6.0 (4.0-8.0)	5.0 (4.0-7.0)	0.0046	0.4785
Positive attitude	5.0 (3.0-8.0)	5.0 (3.0-8.0)	0.8709	
Self-reflective	9.0 (8.0-10.0)	9.0 (8.0-10.0)	0.0831	
Strong clinical skills	7.0 (3.0-10.0)	9.0 (7.0-10.0)		0.0093
Trustworthy	3.0 (2.0-5.0)	4.0 (2.0-6.0)	0.0617	

**Table 9:** Analysis of chief resident experience with order of ranked qualities † Median (IQR); *Estimated using Wilcoxon Signed Rank Test. ** Adjusted for age, gender, and institutional position, and estimated using GLM GLM: generalized linear model

Currently or ever been a chief resident	No	Yes	P-Value, Bivariate*	P-Value, Multivariable Adjustment
Chief Resident Qualities^†^				
Effectively manage multiple demands	4.0 (2.0-7.0)	4.0 (3.0-6.0)	0.6289	
Dependable	2.0 (2.0-4.0)	3.0 (2.0-5.0)	0.1177	
Effectively convey ideas	6.0 (4.0-8.0)	7.0 (5.0-8.0)	0.1800	
Equitable/ Fair	4.0 (3.0-6.0)	5.0 (3.0-7.0)	0.0502	
Good listener	6.0 (4.0-7.0)	6.5 (4.0-8.0)	0.1735	
Open to multiple viewpoints	5.0 (4.0-7.0)	6.0 (4.0-8.0)	0.0138	0.2117
Positive attitude	5.0 (3.0-8.0)	5.0 (3.0-8.0)	0.6228	
Self-reflective	9.0 (8.0-10.0)	9.0 (8.0-10.0)	0.5196	
Strong clinical skills	9.0 (6.0-10.0)	7.0 (4.0-10.0)	0.0008	0.0574
Trustworthy	4.0 (2.0-7.0)	3.0 (2.0-5.0)	0.0238	0.0924

## Discussion

It is reasonable to assume that a broad range of persona qualities are important to CR success and that various stakeholders will deem different qualities as important when selecting CRs, though little data exists to suggest which qualities are valued most highly. This is the largest study to date to survey all stakeholders in CR selection.

From the free-text responses, it was clear that all respondents valued organizational skills. Leadership skills were also important, though this is a somewhat vague quality and was, therefore, not included in the rank-order portion of the survey. There were several interesting differences between the faculty and resident responses. Among residents, the term approachable was the second most common quality identified. Resident responses accounted for 39 of the 44 total responses for this term. Likewise, residents identified being an advocate and good listening more often than faculty. On the other hand, the faculty had a stronger appreciation for the ability to communicate and clinical skills.

These differences in quality valuation are understandable considering the differences in priorities and exposures between faculty and residents. For example, faculty are exposed to residents primarily in the clinical setting, possibly making them more likely to rate CR candidates based on clinical skills, whereas resident peers won’t have as much exposure to a CR candidate’s clinical skills but will more judge a candidate on how approachable they will be or how well they will advocate for the residents.

Another interesting finding from the free-text responses is that the PDs/APDs (the cohort that has worked most closely with CRs) emphasized many different qualities, but no particular quality stood out over others. It is possible that in their experience, CRs can be successful with a variety of qualities, and effectiveness may be determined by the makeup of the CR group rather than by an individual chief.

Interestingly, there was a large difference in the importance of clinical skills between the free-text responses and the rank-order responses. The free-text section (where clinical skills were ranked highly) appeared first in the survey followed by rank-order section (where clinical skills were much lower) on a separate screen. A possible cause of this variance is that clinical skills is one of the first qualities that came to mind in the initial section. This represents a form of availability bias where respondents are more likely to list qualities they encounter frequently and which are most conspicuous. Such bias is known to shape survey responses [14].

In the rank-order section, the qualities of being dependable, trustworthy, and organized (meeting multiple demands) are ranked as most important. In the analysis across groups, the greatest difference between groups was that female respondents placed more importance on effectively managing multiple demands compared to male respondents. The reason for this is unclear; however previous literature suggests it may reflect a difference in traditional domestic responsibilities between genders [15].

A final notable finding is that there was little indicated importance of qualities that might traditionally be associated with academic medicine. In the free-text responses, only 15 respondents identified either ability or an interest in teaching. The term “research” was not used at all. This might suggest that qualities that are important in CRs are those that are broadly applicable in a variety of careers and practice environments, not just in academic medicine.

One significant limitation of this study is the response rate of 43.9%. Participants who chose to respond may value different characteristics than people who did not respond. The study was also only conducted solely within the field of emergency medicine and these results may not be generalizable to other specialties.

## Conclusions

While this study does not provide a clear consensus on the importance of qualities when selecting CRs or the qualities necessary for CR success, this initial set of data could be useful in guiding chief selection processes, CR educational curricula, and CR performance evaluation methodologies. Further research is needed to validate these qualities. This study does demonstrate a variety of opinions on trait importance between respondents (multiple qualities are potentially desirable). From this, PDs might infer that: a) variable qualities are needed depending on the specific role in a specific institution and b) diversity of qualities within a CR group might determine success more than any one quality in any given CR.

## Appendices

Survey

Page 1

Please indicate your position within your institution:

__ R1 resident                                                 __ R2 resident

__ R3-5 resident                                             __ Program director/APD/former PD

__ Other faculty/fellow <5 years from residency

__ Other faculty/fellow >/= 5 years from residency

Please indicate your gender:

__ Male                                                           __ Female

__ Other

Please indicate your age:

__ <25                                                             __ 26-30

__ 31-35                                                          __ 36-40

__ 41-45                                                          __ 45-50

__ >51-55                                                        __ >55

Are you currently or have you ever been a chief resident?

__ Yes                                                              __ No

With which institution are you affiliated?

__ Indiana University                                     __ Southern Illinois University

__ Ohio State University                               __ University of Arkansas

__ University of Texas Southwestern          __ Carolinas Medical Center

__ University of Massachusetts                    __ University of Washington

__ Grand Rapids                                            __ University of Michigan

__ MetroHealth

Page 2

Please list the quality that you feel is most important in a chief resident:

Please list the quality that you feel is the second most important in a chief resident:

Please list the quality that you feel is the third most important in a chief resident:

Page 3

Please rank the following qualities in order of importance (1-10) for a chief resident to possess:

__ Able to effectively manage multiple demands

__ Dependable

__ Effectively conveys ideas

__ Equitable/fair

__ Good listener

__ Open to multiple viewpoints

__ Positive attitude

__ Self-reflective

__ Strong clinical skills

__ Trustworthy
